# NADPH Oxidases: From Molecular Mechanisms to Current Inhibitors

**DOI:** 10.1021/acs.jmedchem.3c00770

**Published:** 2023-08-31

**Authors:** Alessandra Cipriano, Monica Viviano, Alessandra Feoli, Ciro Milite, Giuliana Sarno, Sabrina Castellano, Gianluca Sbardella

**Affiliations:** ^‡^Department of Pharmacy, Epigenetic Med Chem Lab, and ^⊥^PhD Program in Drug Discovery and Development, University of Salerno, via Giovanni Paolo II 132, I-84084 Fisciano, Salerno, Italy

## Abstract

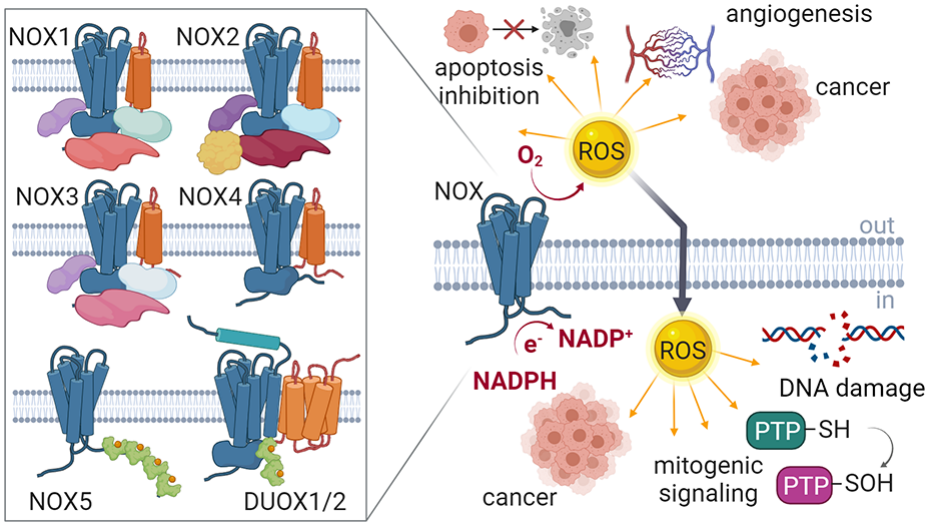

NADPH oxidases (NOXs) form a family of electron-transporting membrane enzymes whose main function is reactive oxygen species (ROS) generation. Strong evidence suggests that ROS produced by NOX enzymes are major contributors to oxidative damage under pathologic conditions. Therefore, blocking the undesirable actions of these enzymes is a therapeutic strategy for treating various pathological disorders, such as cardiovascular diseases, inflammation, and cancer. To date, identification of selective NOX inhibitors is quite challenging, precluding a pharmacologic demonstration of NOX as therapeutic targets *in vivo*. The aim of this Perspective is to furnish an updated outlook about the small-molecule NOX inhibitors described over the last two decades. Structures, activities, and *in vitro*/*in vivo* specificity are discussed, as well as the main biological assays used.

## Significance

- ROS produced by NOXs are major contributors to oxidative damage in pathologic conditions.
- Therefore, blocking the undesirable actions of these enzymes is a therapeutic strategy for treating various pathological disorders.
- The aim of this Perspective is to provide insight on NOX proteins as targets and an overview and update on the current status of NOX inhibitors, including challenges and potential strategic directions for future progress in the field.

## Introduction

**Figure 1:**
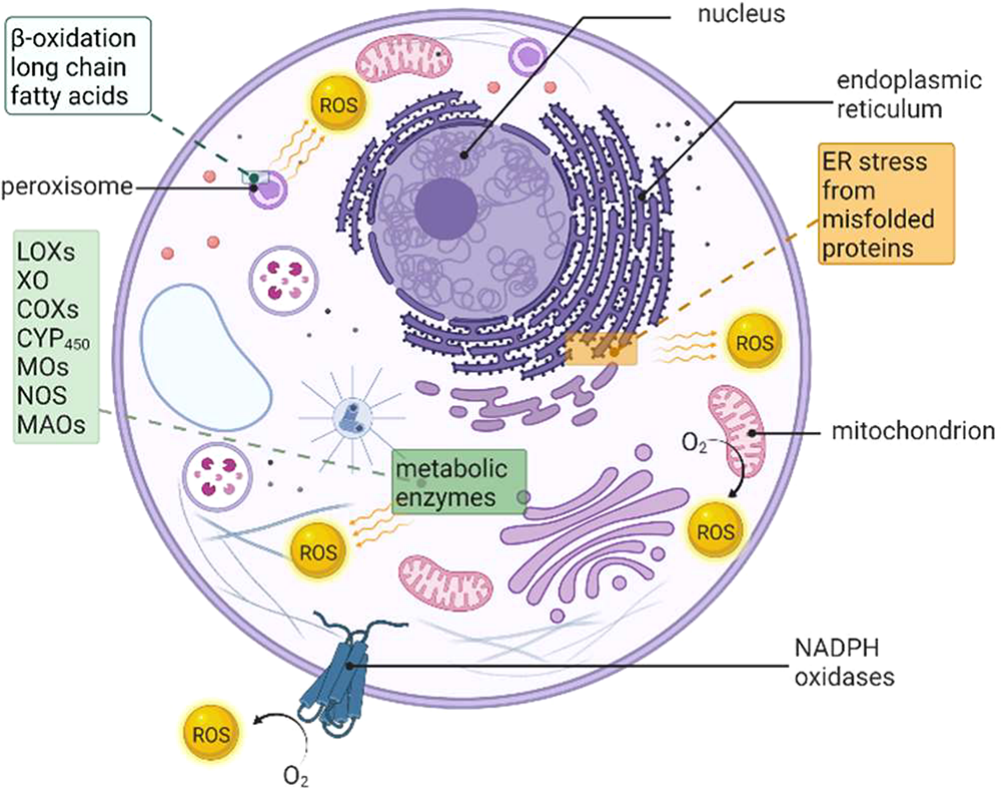
ROS-producing systems. Several enzymes produce ROS as a byproduct of their activities. NADPH oxidases (NOXs) are the only known enzyme family whose sole function is ROS generation. Picture created with BioRender.com.

Reactive oxygen species (ROS) are a group of short-lived intermediates produced by redox reactions or by electronic excitation of oxygen, such as free radicals (i.e., the superoxide anion and hydroxyl radical), as well as nonradical oxidant species (i.e., hydrogen peroxide, H_2_O_2_).^1^ The pivotal role of ROS in different biological processes, spanning from cell homeostasis to inhibition and activation of proteins together with gene transcription, is well established. Several cellular defense systems, such as enzymes that remove oxidants or oxidant scavengers, balance the formation and the reactions of these intermediates. Nevertheless, the increased production of oxidants, coupled with the failure of defense systems, causes an alteration of the proper equilibrium of the cellular redox state leading to the so-called “oxidative stress”.^2^ In this scenario, a cascade of several events lead to different human diseases including fibrosis, cancer, and cardiovascular and neurodegenerative disorders.^3^ Consequently, increasing attention has been paid to endogenous sources of ROS, including the mitochondrial respiratory chain, xanthine oxidase, lipoxygenases, and monoamine oxidase but mainly to nicotinamide adenine dinucleotide phosphate (NADPH) oxidases (NOXs).^4−9^ NOX proteins are a family of enzymes whose distinguishing feature is the production of ROS following specific physiological stimuli (Figure 1).^3^ In this Perspective, we will provide an overview of the structure and function of NOX enzymes, their regulation, and the evidence linking NOX activity to the pathogenesis of various diseases. We also discuss the various NOX inhibitors that have been developed to date, their mechanisms of action, and their potential therapeutic applications.

Finally, we highlight some of the challenges and opportunities in the development of NOX inhibitors as therapeutics and discuss future directions for research in this field.

## Overview on NOX Structures and Regulation

First discovered in immune cells,^10,11^ NOXs are integral enzyme complexes formed by a catalytic core and different subunits, which allow their proper activation and regulation to generate ROS.

To date, seven membrane-crossing enzymes have been identified, namely, NOX1–5 and dual oxidases 1 and 2 (DUOX1 and DUOX2, respectively). Exploring respiratory bursts in neutrophile, the gp91_phox_ catalytic subunit (NOX2) was identified as the first NOX enzyme.^10,12^ Later, other NOX complexes (NOX1,3–5 and DUOX1/2) were discovered and named according to their catalytic domain.^13−16^ In fact, the term “NOX” denotes the transmembrane catalytic domain, but it is commonly used to refer to the entire multiprotein enzyme complex.^17^ Although they share some important features, these enzymes have shown distinct subcellular localization and individual structural and biochemical characteristics, which have implicated them in different pathophysiological processes.^17^ Detailed examination of these aspects is beyond the scope of this Perspective. Nevertheless, the main components involved in NOXs regulation will be described, considering that individual elements may be considered as potential therapeutic targets.

## Architecture of the Catalytic Core

The catalytic core, common to all NOX enzymes, is composed of two distinct domains: the cytosolic dehydrogenase (DH) and the transmembrane (TM) domain. Their architecture has been better elucidated first by X-ray crystal structure deposition of a DH subdomain of *h*NOX2 (PDB ID: 3A1F) and then by the crystal structure of the DH (PDB ID: 5O0X) and TM (PDB ID: 5O0T) domains of NOX5 from *Cylindrospermum stagnale*.^18^ Recently, two separated studies that reported the cryo-EM structures of DUOX1/2 helped gain further structural information.^19,20^

**Figure 2:**
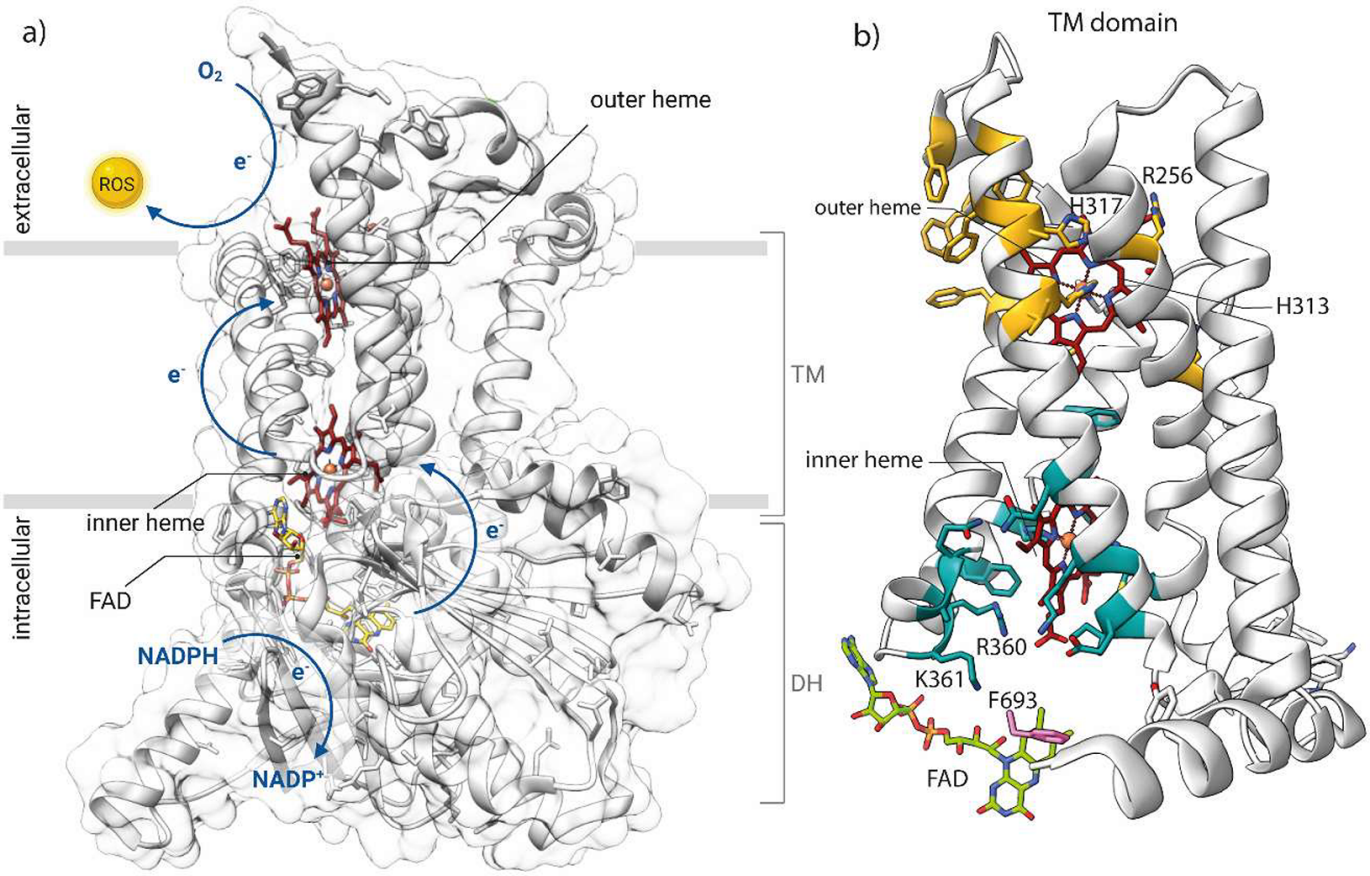
Representation of the electron transfer in the catalytic core of NOX enzymes (a) built from the structures of the DH (PDB 5O0X) and TM (PDB 5O0T) domains of NOX5 with ChimeraX. Heme groups are depicted in dark red and FAD cofactor, in yellow. The focused view of the TM domain (b) highlights the most involved amino acid residues. The top part shows the oxygen binding and reacting site as a small cavity exposed to the external environment, lined by the propionate 7 of the outer heme and the side chains of conserved residues R256, H317, and H313 (in golden rod). The bottom part of the TM domain contains its interacting surface with the DH domain and outlines the highly conserved R360 in the D loop (in dark cyan) near propionate 6 of the inner heme. In proximity of the interdomain interface lies a conserved residue of the DH domain C-terminus, F693 (hot pink). This residue functions as a toggle-switch gating access to the NADPH substrate to initiate catalysis. In close proximity, there is positively charged K361 (dark cyan). To avoid confusion with the residues of the oxygen-reacting site, FAD is depicted in chartreuse.

The DH domain encloses an N-terminal region that binds the flavin adenine dinucleotide (FAD) cofactor and a C-terminal lobe that binds the NADPH. On the other hand, the TM domain consists of six transmembrane helices and two prosthetic heme groups positioned in a pocket formed by helices 2–6. Both heme groups are hexacoordinated, interacting with two pairs of histidines in helix 3 and 5. Considering their orthogonal orientation with respect to the plane of the lipid bilayer, one heme group is near the cytosolic side (inner heme, Figure 2), while the other is oriented toward the extracytoplasmic side (outer heme, Figure 2).^3,21^

Through these domains, NOXs are able to transfer electrons, supplied by NADPH, across biological membranes, following a mechanism summarized as follows and depicted in Figure 2. In the first step, two electrons are transferred from NADPH to FAD, reducing it to FADH_2_. Later, the electrons are moved from the inner to the outer heme and, finally, to the oxygen on the extracellular side and, thus, reduced to superoxide anion, which can be protonated and reduced to form H_2_O_2_ or other ROS species.^22^

Mattevi and co-workers found that the O_2_-binding site is a small cavity, containing a highly ordered water molecule, positioned above the outer heme. This pocket is surrounded by the heme propionate 7 and three closely conserved residues: H317, iron-coordinating H313, and R256 (Figure 2b). The latter, due to its positive charge, can electrostatically increase superoxide production.^18^

## NOX Complexes: Subunits and Regulation Mechanisms

**Figure 3:**
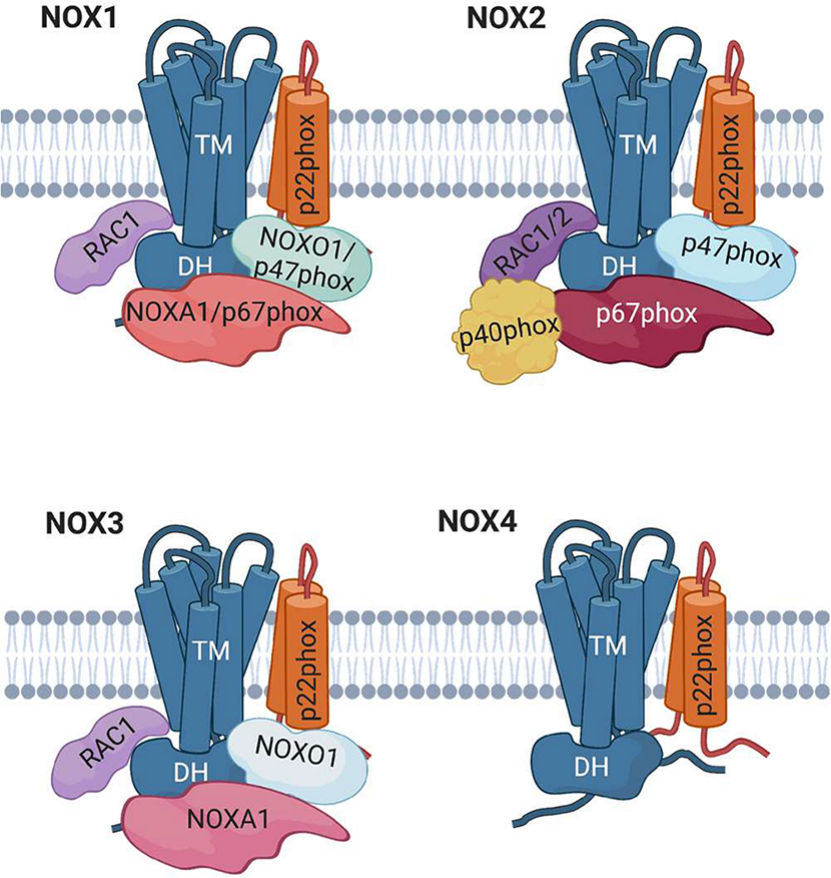
Cartoon representation of the NOX1–4 enzymes.

Although they share a very similar catalytic core architecture, the subunits that constitute the multiprotein complex of individual NOX proteins are different. By distinct mechanisms, these subunits finely regulate the activation of each enzyme to produce ROS. Overall, NOX1–4 activation relies on the association of the catalytic subunit with the p22_phox_ subunit. In contrast, activation of NOX5 and DUOX1/2 does not require the p22_phox_ subunit but depends on a calcium-dependent activation mechanism (Figure 3).^17,23,24^

### NOX1–4

The enzymatically active complex of NOX1–4 isoforms (Figure 3) involves the formation of a heterodimer between the catalytic core and the transmembrane 22-kDa subunit p22_phox_, forming the so-called Cytochrome b_558_.^25^ In particular, several domains of p22_phox_ are involved in the proper maturation and expression on the cell surface of the catalytic subunit.^26^ In resting phagocytes, both subunits are found in the membranes of specific granules and/or secretory vesicles and fuse with the plasma membrane only upon specific physiological stimuli.^27^ Nevertheless, the formation of the heterodimer between the catalytic subunit and p22_phox_ is not sufficient for full enzymatic activity. In fact, at least for NOX1 and NOX2, the proper enzymatic activation requires the assembly of Cytochrome b_558_ with other cytosolic subunits to the plasma membrane, as well as the activation of the low molecular weight GTP-binding protein (i.e., RAC1/2).^23^ The tight and requisite association between p22 and NOX2 was explained by the recent solution of the structure of the inactive heterodimeric NOX2–p22 core complex bound to a selective anti-NOX2 antibody fragment.^28^ The structure showed that the p22 subunit adopts a four-helix transmembrane domain fold that binds the catalytic NOX2 subunit across three extensive and conserved interface regions, which include well-resolved membrane lipids. Moreover, the highly ordered extracellular loops of NOX2 form a glycan-decorated cap atop the outer heme that is conserved across the NOX1–4 subfamily and not featured in NOX5, thus rationalizing the lack of binding of p22 to NOX5.

NOX2 was the first enzyme discovered and is found mostly in phagocytic cells but also in other tissues like kidney, fibroblasts, osteoclasts, thyroid.^29,30^ The other NOXs have been studied based on homologies and/or differences with it. The NOX2 complex is formed by three cytosolic subunits, termed cytosolic phox (phagocytic oxidase): p47_phox_,^31,32^ p40_phox_,^33^ and p67_phox_.^34^

p47_phox_ is an adaptor protein whose main function is to bind both the p22_phox_ and p67_phox_ subunits by bringing them closer. Specifically, p47_phox_ contains two SH3 domains (SRC homology 3) and a proline–proline–arginine-containing region that acts as an autoinhibitory region (AIR).^35^ The inactive state of the subunit is maintained by intramolecular interactions between the AIR and SH3 domains, blocking their translocation and anchoring them to the membrane. Following phosphorylation, AIR undergoes a conformational change that exposes SH3 domains in tandem for binding to p22_phox_. Sequentially, this interaction initiates translocation of the cytoplasmic subunits to the membrane and the formation of the active complex.^36^ The p67_phox_ subunit contains an activation domain that binds the gp91_phox_ subunit and its own NADPH binding site, which allowed the direct transfer of electrons from the NADPH to the FAD center of the catalytic subunit. In addition, the p67_phox_ subunit also contains a RAC-binding domain (Figure 3).^27,37^ RAC is a key component for the assembly of an active NADPH oxidase, interacting with p67_phox_ in a 2-fold way, both by bridging this subunit closer to the membrane and by inducing its conformational change in a more active form. It has been shown that this association is enhanced when RAC is complexed with the Rho GDP dissociation inhibitor (Rho-GDI), usually preventing the GDP/GTP exchange reaction. In NOX enzymes, Rho-GDI stabilizes RAC in an active conformation, even in the GDP-bound state. First, RAC interacts with p67_phox_ to form a low-affinity ternary complex RAC-GDP/Rho-GDI/p67_phox_. Then, GDP/GTP exchange on RAC generates a higher affinity conformation in a GTP-bound form.^38,39^ Recently, Heo and co-workers showed that RAC has an important role in NOX2 autoactivation. In fact, superoxide production by NOX2 triggers redox-sensitive RAC, which in turn, further activates NOX2, amplifying the positive feedback loop between RAC and NOX2.^40^

Overall, very similar mechanisms regulate the activation of NOX1, which shares almost 60% sequence identity with NOX2. This enzyme is mainly expressed in the colon but also in prostate, uterus, placenta, osteoclasts, and vascular cells.^13,41,42^ Like NOX2, NOX1 dimerizes with the p22_phox_ subunit and is activated by RAC1 and by the cytosolic factors NOXO1 and NOXA1 (for NOX organizing and activator of protein 1, respectively), homologous to the p47_phox_ and p67_phox_ NOX2 subunits, respectively. Like p47_phox_, NOXO1 interacts with p22_phox_ via the SH3 domain. However, this subunit lacks the AIR region, thus activating NOX1 in the absence of cell stimulation. This activation occurs through interactions of NOXO1 with characteristic lipids, which colocalize it with NOX1 in the membranes of resting cells, constitutively interacting with them.^43^ Despite the low amino acid identity (approximately 28%), NOXA1 has a domain structure very similar to that of p67_phox_, containing different domains that allow binding both to NOXO1 and RAC.^44^

The NOX3 enzyme has been specifically localized to the inner ear contributing to the proper development of otoconia crystals of the vestibular system.^45^ Moreover, it has localized in fetal tissues, mostly in kidney, with lesser expression in other fetal tissues including liver, lung, and spleen.^29^ Differently from NOX1 and NOX2, NOX3 is active alongside the interaction with the p22_phox_ subunit.^46^ Nevertheless, enzyme activity has a high degree of flexibility in its regulatory mechanisms, employing combinations of different subunits.

NOXO1 is able to activate NOX3 alone and in the absence of NOXA1, probably by inducing an active conformation in the catalytic subunit even in the absence of a protein containing an activation domain.

In addition, NOX3 can be slightly activated by p67_phox_, and this effect can be enhanced by the addition of p47_phox_, which alone cannot activate the enzyme.^47^ NOX4 shares only 39% identity with NOX2. This enzyme was first found in the kidney but also in other cells such as osteoclasts, endothelial and smooth muscle cells, fibroblasts, keratinocytes, and neurons.^48−51^ Despite the interaction with the p22_phox_ subunit for ROS generation, the activity of NOX4 does not require cytosolic subunits to be constitutively active. The enzymatic activity is probably regulated by its cellular localization and activating factors. An interesting work by Block and co-workers showed that NOX4 activity in the mitochondrial compartment is regulated by adenosine triphosphate (ATP) levels. During normal respiration, the ATP produced binds NOX4 in a specific domain, keeping ROS production low. A lowering of ATP levels, due to various cellular events such as cancer, lead to the activation of NOX4.^52^ Together with DUOX1/2, NOX4 primarily generates hydrogen peroxide. Brandes and co-workers identified a large E-loop of the enzyme as an essential structural feature for this process, involving specific cysteine and histidine residues within the loop. Although the molecular mechanism is still not well-defined, it has been hypothesized that the enzyme may function as a dioxygenase or have endogenous superoxide dismutase activity, using the above-mentioned residues in either case. Another hypothesis is the creation of a “cage” in the E-loop that accelerates the rate of spontaneous dismutation by involving cysteine and histidine residues as proton donors.^53^

### NOX5 and DUOX1/2

**Figure 4:**
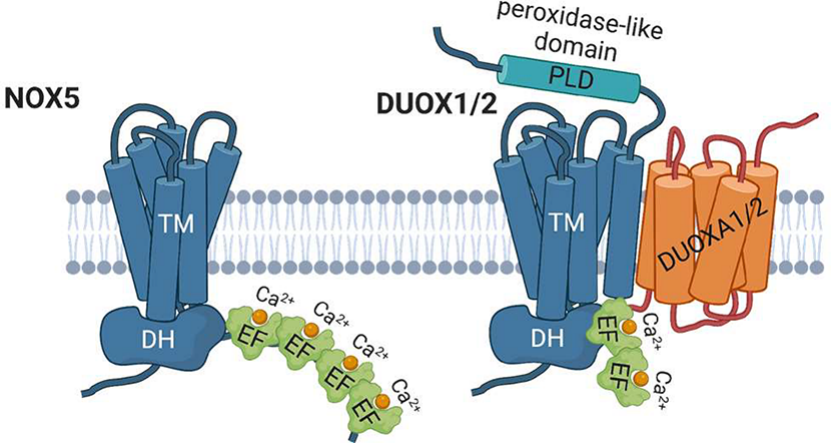
Cartoon representation of the NOX5 and DUOX1/2 enzymes.

Conversely, NOX5 and DUOX1/2 do not interact with the p22_phox_ subunit, and their activation involves different processes. NOX5 (Figure 4) is a monomeric protein expressed in several tissues, mainly in the spleen and testis but also in vascular tissue, cells of the gastrointestinal tract, reproductive systems, and fetal organs.^15,29,54^

The distinguishing feature of NOX5, despite sharing the same structural organization as other NOXs, is the presence of an *N*-terminal EF-hand domain that allows calcium-dependent regulation. The EF-hand domain consists of an *N*-lobe and an *C*-lobe, each containing two EF-hand motifs. The *N*-lobe has a well-defined tertiary structure, while the *C*-lobe acquires an ordered structure only in the presence of calcium ions for which it has a higher affinity.^55^ Increased calcium ion levels lead to a conformational change in the EF domain that allows the *C*-lobe to interact with the regulatory EF-hand-binding domain (REFBD), an autoinhibitory element, within the DH domain. Following this interaction, the REFBD domain is removed from the catalytic site, thus activating the enzyme.^56^ Interestingly, mutations of conserved residues in the predicted EF binding region of the DH domain, as well as mutations of the corresponding residues in the DH domain of NOX2, cause inactivation of the enzyme. This suggests that the DH region has an important regulatory role not only in NOX5 but in all NOX enzymes.^57^ Other pathways also seem to be involved in NOX5 activation and regulation.^58−61^ Overall, these mechanisms involve both post-translational modifications and protein–protein interactions. NOX5 phosphorylation was found to have an important role in NOX5 regulation. The phorbol 12-myristate 13-acetate (PMA) enables enzyme activation at lower levels of intracellular calcium, enhancing the phosphorylation of NOX5 at specific serine and threonine residues.^61^ Further studies revealed that this phosphorylation is mediated mainly by the α isoform of protein kinase (PKCα). Other PKC isoforms involved are PKCδ and PKCε, which may influence superoxide release through indirect mechanisms.^60^ Besides phosphorylation, other post-translational modifications can regulate NOX5, mainly by reducing its activity. For example, it has been found that methionine and cysteine residues within the protein can be oxidized, thereby decreasing Ca^2+^ binding and, consequently, the activity of the protein. Similarly, *N*-nitrosylation on cysteine residues can reduce oxidase activity in the presence of exogenous and endogenous nitric oxide. Both mechanisms may serve as a possible defense against excessive ROS generation by NOX5.^62,63^ Regarding the regulation mediated by protein–protein interactions, several proteins have been identified as NOX5 binders. In this context, calmodulin is able to interact with a binding site in proximity to that of NADPH. It has been shown that calmodulin has no effect when calcium concentrations necessary for enzyme activation are reached. However, when ion levels are low, calmodulin contributes to the activation of NOX5, increasing its sensitivity to calcium.^64^ Moreover, a recent study suggested that calmodulin may contribute to the stabilization of the dimeric form of the DH domain and that the oligomeric states of NOX5 may enhance ROS generation.^65^ The binding of NOX5 to heat shock protein 90 (Hsp90) through its *C*-terminal domain proved to be relevant. This interaction plays an important role in stabilizing the DH domain of NOX5 and preventing the formation of active NOX5 oligomers. Binding to calcium ions leads to conformational changes that appear to displace Hsp90 and remove its autoinhibitory interaction.^66^ NOX5 could also be regulated by hydrogen peroxide and the nonreceptor tyrosine kinase c-Abl. Indeed, it has been shown that H_2_O_2_ can stimulate its own production by simultaneously promoting the low calcium influx and activation of c-Abl through phosphorylation. These events promote translocation to the membrane and oligomerization of c-Abl, which can thus directly or indirectly interact with NOX5, stimulating its activity and potentially enhancing its sensitivity to low calcium concentration. Activation of NOX5 promotes the production of superoxide anion and hydrogen peroxide, which can amplify the first step of this regulatory pathway.^67^

In analogy with NOX5, DUOX1 and DUOX2 (Figure 4) are tightly regulated in a calcium-dependent manner.^16,68^ These enzymes are highly expressed in thyroid tissue^16^ and, like NOX4, catalyze the direct production of H_2_O_2_. Besides the common catalytic core, DUOX1/2 contains an EF-hand domain but, unlike NOX5, with two EF-hand motifs instead of four and a N-terminal extracellular peroxidase-homology domain. The activity of each enzyme is closely linked to interactions with auxiliary proteins called double oxidase maturation factor (DUOXA1 and DUOXA2, respectively), required for the proper maturation, subsequent migration to the plasma membrane, and full enzymatic activity of DUOX1/2, forming a stable heterodimer.^69,70^ A recent study showed the structure of the *h*DUOX1 complex in both the high-calcium and low-calcium state. In the first case, the DH and TM domains are properly oriented for redox reaction through multiple interdomain interactions. On the other hand, in a low-calcium state, these interactions change, reducing the electron transfer efficiency.^19^ Moreover, like NOX5, the enzymatic activity of DUOX1/2 is regulated by different mechanisms. For example, DUOX1 is positively affected by the cAMP-dependent protein kinase A, while the activity of DUOX2 is enhanced by protein kinase C at very low concentrations of PMA.^71^

## NOXs and Diseases

**Figure 5:**
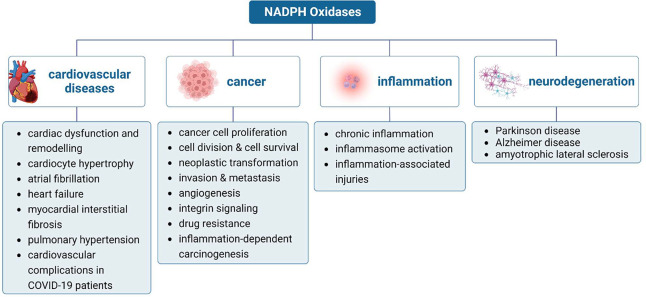
Main pathophysiological consequences of NOX-mediated ROS generation.

ROS are involved in a wide range of pathways, and consequently, NOX proteins are implicated in various pathological disorders (Figure 5). Hereafter, we report the description of the main pathophysiological consequences of the NOX-mediated ROS generation, focusing on cardiovascular and neurodegenerative diseases, immune system, and cancer.^72^

### NOXs in Cardiovascular Diseases

NOX enzymes are the main source of ROS in cardiovascular events, resulting in oxidative stress that is related to dysfunction of cellular signaling mechanisms sensitive to changes in redox states, modification of key regulatory proteins, and direct damage of cellular molecules, such as DNA, proteins, and lipids.^73−75^ Under physiological or pathological conditions, ROS species can alter various processes, such as endothelial function and vascular tone.

In the vascular system, NOX activation is mediated by angiotensin II (Ang II), a major vasoconstrictor of the renin-angiotensin system (RAS), through several mechanisms both at gene, transcriptional, and post-transcriptional levels. The ROS produced affects several downstream Ang II signaling targets and regulates Ang II receptors.

Under pathological conditions (i.e., hypertension, diabetes, and atherosclerosis), Ang II stimulates NOX hyperactivation, which in turn promotes numerous processes, including the synthesis of proinflammatory mediators, expression of adhesion cells, increased vascular permeability, and calcification.^76−79^ Several studies in animal models have confirmed the role of NOX activation in the development of Ang II-induced hypertension. For example, global knockout of NOX1 or NOX2 has been shown to protect against Ang II-induced hypertension, and higher mRNA levels of NOX1, NOX2, and NOX4 were found in the aortas of Ang II-infused animals.^80−84^ In addition, it has been reported that NOX5 is upregulated in cultured human endothelial cells.^85^

Moreover, NOX-derived ROS contribute to the activation of other oxidase systems, such as dysfunctional mitochondria and the uncoupled endothelial nitric oxide synthase (eNOS), and the production of superoxide rather than nitric oxide (NO).^73−75,86^

NOX-driven uncoupling of eNOS mediates hypertension, and, in particular, it is a causal factor for the development of abdominal aortic aneurysm (AAA): genetic knockout of NOX1, NOX2, p47_phox_, or NOX4 prevented formation of AAA, reducing abdominal aortic expansion and restoring eNOS coupling activity.^87^ Moreover, ROS avidly react with and inactivate NO and, in the process, produce highly reactive and cytotoxic products.^88^

The role of NOX isoforms, especially NOX2 and NOX4, has been studied in the development of cardiac hypertrophy, in which these enzymes activate several downstream pathways.^89−91^ In addition, some evidence correlates NOX proteins with cardiac arrhythmias, associating these enzymes with elevated ROS production.^92,93^

NOX2 and NOX4 play an important role in ischemia-reperfusion (IR) damage.^94^ It has been proposed that deletion of NOX2 or NOX4 and, consequently, a slight reduction in oxidative stress may be involved in cardioprotection against IR injury. On the other hand, marked reduction of oxidative stress (e.g., through combined knockout of NOX2 and NOX4) increases cardiomyocyte death.

In general, a proper balance of ROS levels is needed, because basal or elevated levels can give completely opposite outcomes, ranging from cardioprotective effects to myocardial infarction.^88,95,96^ Interestingly, studies in transgenic animals with the human isoform of NOX5 have shown that, differently from that of IR-induced infarction, the size of cerebral infarction after stroke increases because of ROS rising, probably due to a loss of the blood–brain barrier.^97^

Another important pattern of cardiovascular disease in which NOX proteins are implicated is atherosclerosis. Indeed, NOX-derived ROS induces low-density lipoprotein (LDL) oxidation, a crucial event in the early stages of atherogenesis. However, individual isoforms mediate different effects. Deletion of NOX1 and NOX2 in Apoe^–/–^, a widely used model of atherogenesis, reduced superoxide production and lesion formation in the aorta.^98−100^ Conversely, a protective role has been attributed to NOX4 in atherosclerosis, probably due to the beneficial effects of H_2_O_2_ produced by the enzyme, that should lead to inhibition of inflammation.^101,102^

Several recent studies also suggested the pivotal role of NOXs in cardiovascular complications in COVID-19 patients. Specifically, compared to controls, SARS-CoV-2 infected patients displayed overactivation of NOX2, which was more marked in subjects with thrombotic complications, indicating a role in thrombotic-related ischemic events.^103^ More recently, the induction of NOX2 and NOX5 in the cardiac microvascular endothelium was also reported, even if the exact roles of NOXs in the pathogenesis of COVID-19 remain to be elucidated.^104^

### NOX and Neurodegeneration

To maintain homeostasis and neuronal cell function, the human nervous system consumes about 20% of the amount of oxygen used by the body, causing a large production of ROS. As a result, it could be very sensitive to oxidative stress, which in turn plays a central role in neuroinflammation and neurodegenerative diseases, such as Parkinson’s disease (PD), Alzheimer’s disease (AD), and amyotrophic lateral sclerosis (ALS).^105,106^ In this context, the role of NOXs as a source of ROS, especially NOX2, being the main isoform in the brain besides NOX1 and NOX4, has been intensively investigated.^107−109^ In experimental animals and in humans, the activation of NOX2, whose overexpression in endothelial cells leads to brain oxidative stress and DNA damage, has been associated with aging-related rarefaction of brain capillaries, loss of neurons, and locomotor disorders, all key aspects of neurodegenerative diseases.^110^

Oxidative stress is believed to be the common underlying mechanism leading to determinants in PD such as α-synuclein misfolding and/or aggregation, neurotoxicity, and degeneration of dopaminergic neurons.^111^ Nuclear localization of NOX1 and its RAC1-mediated activation to generate ROS have been implicated in the degeneration of nigrostriatal dopaminergic neurons in animal models of PD.^112,113^ During PD, elevated levels of NOX4 have been detected in hippocampus, which directly cooperates with neuroinflammatory cytokines through mitochondrial dysfunction in hippocampal astrocytes.^114^ The role of NOX2 in both microglia and neuronal cells, whose activation and/or oxidative damage were found in the substantia nigra of PD patients, was also studied. Overall, several *in vitro* and *in vivo* studies have demonstrated that microglia activation and dopaminergic neurodegeneration implicated in PD are propagated through microglial NOX2 activation, following both exogenous and endogenous stimuli.^115−117^ Activation of neuronal but not microglial NOX2 has been observed in acute and subacute PD models, suggesting that neuronal NOX2 may play a primary role in the early stages of the disease.^111^ Furthermore, in addition to the basal expression of NOX1, NOX2, and NOX4 in neurons, only NOX2 is upregulated under inflammatory conditions. Gao and co-workers proposed a model in which microglial NOX2 activation increases the production of superoxide and H_2_O_2_. These species raise neuronal intracellular ROS levels, which further activate NOX2, according to a positive feedback mechanism between ROS production and increased neuronal NOX2. A cascade of events follows, including the release of pro-inflammatory factors by activated microglia, which worsens oxidative stress in neurons, causing their damage.^118^

An important connection has also been described between NOX proteins and the α-synuclein, whose misfolding or abnormal aggregates have been related to several neurodegenerative diseases and represents a hallmark of PD.^119^*In vitro* and *in vivo* PD models highlighted the role of NOX1 in modulating α-synuclein expression and aggregation in dopaminergic neurons. Specifically, exogenous induced oxidative stress raises α-synuclein aggregation levels, which can be restored by NOX1 knockdown.^120^ PD patients, who exhibit α-synuclein accumulation, have enhanced NOX4 activity, the expression of which increases from asymptomatic patients to those with established PD.^121^ Moreover, it has been demonstrated that mitochondrial ROS activate neuronal NOX2, resulting in α-synuclein oligomerization, thus amplifying its downstream mitochondrial dysfunction.^111^ All of this evidence emphasizes the role of oxidative stress in PD, paving the way to new therapeutic approaches in this neurodegenerative disease.

NOX2-induced oxidative stress plays an important role in the vascular neurotoxic amyloid β (Aβ) aggregates, a hallmark of Alzheimer’s disease.^122−124^ Overall, Aβ induces NOX-derived ROS in neuronal and non-neuronal cell cultures, which in turn, leads to a loss of endothelial cell–cell interactions, loss of the blood–brain barrier, and disruption of tight junctions (TJs) in the brain microvascular endothelium. For example, NOX2-activated microglia have been found to surround Aβ-loaded capillaries in which a loss of TJ proteins has been observed.^125^ In AD mice models, deficiency of the NOX2 catalytic subunit has been shown to prevent oxidative stress, cerebrovascular dysfunction, and behavioral deficits without reducing brain Aβ levels or amyloid plaques.^126^*In vitro* and *in vivo* experiments have demonstrated the role of NOX2-derived ROS as major redox signaling pathways in mediating the microglial response to stimulation with Aβ42, the predominant Aβ species found in amyloid plaques of AD patients.^127^ Zilberter and co-workers found that NOX2 activation by oligomeric Aβ42 causes cerebral glucose hypometabolism, hippocampal network hyperactivity, and neuropsychiatric-like behavioral disorders in mice.^128^ Also, NOX4 is upregulated in AD patients. It has been shown that neuronal knockdown of NOX4 gene in mice resulted in a reduced accumulation of pathological tau proteins, whose aggregates characterize several neurodegenerative diseases including AD. Moreover, a reduced neurotoxicity and cognitive decline have been observed in neuronal-targeted NOX4 knockdown, supporting the direct involvement of NOX4 in accumulation of tau proteins.^129^ Higher NOX4 levels have also been found in astrocytes of the cerebral cortex from AD patients, leading to disruption of mitochondrial metabolism and oxidative stress. The latter promotes lipid peroxidation in astrocytes and, consequently, ferroptosis, a type of programmed cell death that is dependent on iron and the lipid peroxidation, both involved in neurodegenerative diseases.^130^

Overproduction of NOX-derived ROS also contributes to the onset and progression of amyotrophic lateral sclerosis (ALS). Also, in this disease, higher expression levels of NOX2 were found both in the spinal cords of ALS patients and in transgenic mice used as ALS models. NOX2 deletion improved survival and retard neurodegeneration. In addition, NOX-derived oxidant products can damage proteins located on motor neurons, where receptors for IGF1, a trophic factor known to promote motor neuron survival, are present. The IGF1 signaling pathway can be disrupted as a result of a NOX-dependent mechanism that causes oxidative changes in receptors.^131^ Transgenic mice for superoxide dismutase-1 (SOD1), a well-known hallmark of ALS, showed NOX1/NOX2-dependent oxidative stress that has been linked to the progression of motor neuron disease. Disease progression and improved survival have been lowered by deletion of either NOX1 or NOX2, even if NOX2 removal increased survival rates 50% more significantly than NOX1 deletion.^132^ Based on these findings, in 2016, a study aimed to evaluate NOX2 activity in a series of ALS patients was performed. NOX2 activity was assessed in peripheral blood cells from ALS patients and matched controls. In both cases, the authors found that NOX2 activity was not significantly different and was independent of sex, age, or disease duration. However, patients with reduced NOX2 activity also had a marked improvement in survival, independently of other known prognostic factors, with a 7.6-fold risk of death.^133^

### NOXs in Immune System

Immune host defense constitutes only one of the many physiological functions in which ROS are involved. As enzymes are dedicated to the production of these reactive species, the NOX family directly contributes to this activity.

While the production of superoxide by NOX was initially hypothesized as the only relevant process responsible for the bacterial destruction, it has been demonstrated that ROS-mediated elimination is the result of a complex cooperation between different mechanisms,^134^ as well as of other ROS-independent killing machineries supported by NOX enzymes.^135,136^

The plentiful attempts made, in recent decades, to clarify the mechanisms involved in immune defense during phagocytosis have allowed researchers to better figure out the whole NOX family. In fact, individuals with chronic granulomatous disease (CGD), a condition characterized by increased sensitivity to infections, show mutations in NOX2, the main source of ROS in polymorphonuclear leukocytes (PMNs).^137−140^

In the case of bacterial infection, several chemotactic compounds such as interleukin 8 and formyl peptides released by mitochondria or bacteria^141^ are responsible for regulating the migration of PMNs and the activation of NADPH-oxidase. In particular, the destruction of internalized pathogens is due to a series of subsequent events, such as phagosome formation, fusion of cytosolic granules containing bactericidal peptides and proteins with the phagosome, and assembly of the NOX2 complex with consequent production of ROS.^142^ In addition to the direct contribution to the elimination of pathogens, ROS produced by NOXs selectively disable bacterial virulence factors as an innate host defense mechanism. In some bacteria, such as *Staphylococcus aureus*, HOCl resulting from the superoxide anion generated by NOX oxidizes and inactivates the quorum sensing peptides responsible for the virulence of microorganisms.^142^

Although NOX2 is the main member of NOX enzymes in innate immunity, it is not the only NADPH oxidase involved in pathogen response.^143^ In fact, the expression of DUOX1/2 in the mucous membranes of the airways is associated with the production of H_2_O_2_ from which lactoperoxidase generates compounds with microbiocidal action through the oxidation of thiocyanate and iodide.^144^

Several studies in *Drosophila* have allowed further documentation of the role of DUOX1/2 in the host defense: in flies, the silencing of the Duox gene was responsible for increased infections by intestinal microbes and subsequent mortality. These effects were completely reversed by the reintroduction of DUOX1/2, thus confirming its key role in gut immunity.^145^

NOX1 also participates in mucosal immunity, as suggested by its localization in the colon and its ability to partially replace NOX2.^146^ In addition, an interaction between NOX4 and toll receptor 4 (TLR4), a pathogen recognition receptor,^147^ has been demonstrated. This functional link results in the activation of transcription factors (e.g., NF-kB) involved in the innate immune response.^148^

### NOXs in Cancer

ROS intracellular production has been experimentally associated with the development of cancer.^149^ These reactive species are able to induce DNA damage by the introduction of several modifications in bases and sugars, the generation of cross-linking between DNA and proteins, and the production of breaks in the DNA strand. Since the low redox potential of guanine (G), its oxidation to 8-oxo-dG represents one of the most common kinds of damage.^150^ Moreover, the similarity of this product with thymine (T) makes G-to-T transversions extremely common, representing the major somatic mutation in lung, breast, ovarian, gastric, and colorectal cancers.^151^ ROS production is also associated with the perturbation of signaling pathways that affects the growth and evolution of cancer. In fact, ROS have been demonstrated to react and directly inhibit the activity of protein tyrosine phosphatases (PTPs), through the oxidation of the sulfuric atom of the catalytic site cysteine and the formation of the so-called “sulfenamide modification” with the nitrogen of the amide backbone of the following residue.^152^ The inactivation of these enzymes plays a key role in the activation of receptor protein tyrosine kinases and, consequently, in the differentiation, proliferation, and survival of malignant cells.^153,154^ In this context, NOX-derived ROS are logical contributors to these phenomena, although the exact function of these enzymes in cellular alteration remains unclear.

On the other hand, it has been largely shown that cancer cells generate huge amounts of ROS and are responsible for overexpression of NOX enzymes as well as underexpression of antioxidant defense systems.^155^

**Figure 6:**
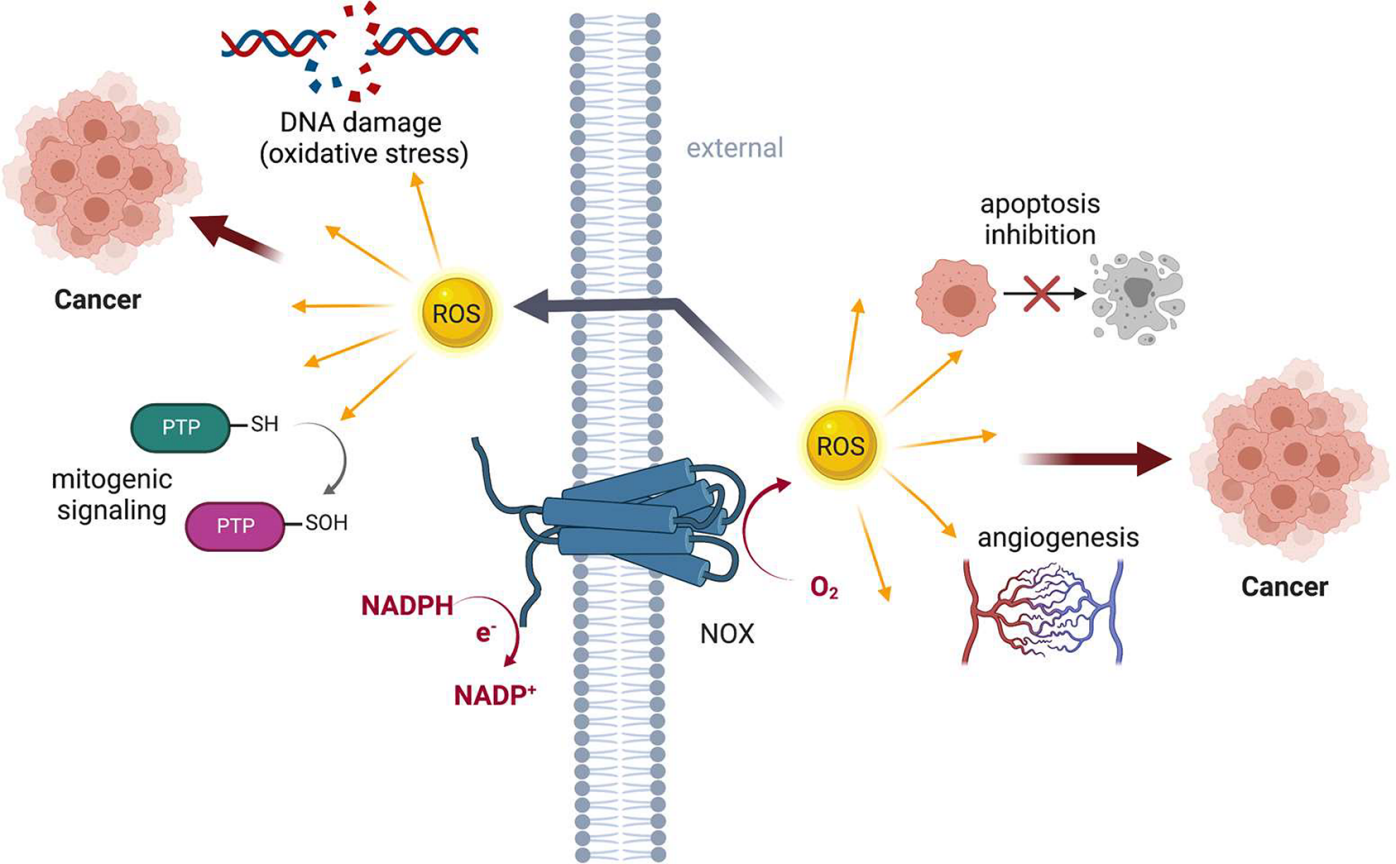
Tumorigenic signaling by NOX enzymes.

The identification of NOX enzymes has offered a better understanding of the ROS signaling function in cancer progression (Figure 6). Therefore, understanding the roles played by NOX isoforms in tumor development and progression has become of emerging interest in recent years.

Recently, it has been proven that NOX1-generated ROS play a vital role in the proliferation and invasions of colon cancer cells. It has also been seen that the knockdown of NOX1 in HT-29 cells inhibited MAPK signaling and blocked the G1/S phase.^156^ NOX1 is also reported to take part in Ras-induced VEGF expression and angiogenesis: growth factors such as EGF are responsible for the stimulation of tyrosine kinase, inducing NOX1 expression.^157^

An interesting study demonstrated the correlation between expressions of the NOX regulatory subunits and BRCA1 gene in ovarian cystadenocarcinoma, lung adenocarcinoma, and breast invasive carcinoma, suggesting that the high expression of regulatory subunits of NOX1 and NOX4 are related to the downregulation of BRCA1 gene expression, and these events may be associated with the progression of malignancy.^158^

It was also found that NOX1 and its activating protein p67_phox_ were upregulated in E2-induced tumors in rats and in human breast tumors that are estrogen receptor-positive (ER+). In fact, the oxidative stress induced by NOX1 is responsible for the downregulation of the antiapoptotic protein surviving and for the subsequent initiation of ER+ breast tumor formation.^159^

It has been proven that tumor metastasis is strictly related to chemoresistance and that drug resistance during chemotherapy is increasing in cancer patients. Korkina and co-workers suggested that one of the hallmarks of the drug resistance was the alteration of the redox homeostasis.^160^ The high expression of NOX1 promotes intracellular ROS generation, thus activating the HIF-1α/MDR1 pathway to speed up the chemoresistance in gallbladder cancer cells.^161^ Moreover, HIF-1α prompts the expression of P-glycoprotein, which incites chemoresistance in prostate cancer cells.^162^ Therefore, all of these findings confirm that NOX1 is closely related to the development of drug resistance in cancer.

As with NOX1, it has been demonstrated that the role of NOX2 in cancer development is related to angiogenesis. Indeed, this isoform is the major source of ROS generated by VEGF and AngI, two elements involved in the growth of tumor vessels.^163^

ROS may also be correlated to the silencing of the immune response to cancer: the myeloid-derived suppressor cells (MDSC), responsible for ROS-dependent immune suppression in tumors, showed high ROS levels in different kind of tumors.^164,165^ Notably, the lack of NOX2 activity has been related to the MDSC loss of the ability to suppress the response of T cells, thus suggesting that NOX2 is strictly associated with ROS-induced immune suppression by MDSC. Moreover, a noteworthy report revealed that the potent carcinogen PMA stimulates NOX2 expression. In particular, it is connected to the invasion of colon cancer cells through elevated expression of the matrix metalloprotease MMP-7.^166^ In addition to its role in angiogenesis and cardiovascular diseases, NOX4 has been reported to take part to genomic instability, cell death, and cancer.^167^ In fact, NOX4 is the most frequently expressed NOX isoform in several malignancies such as neuroepithelial tumors, human melanomas, and lung, renal, colorectal, gastric, pancreatic, and ovarian cancers. Beyond its overexpression in human tumors, NOX4 appears as a crucial mediator in cell transformation and tumor growth, a function emphasized by the finding that it is able to activate various signaling pathways and to mediate metabolic plasticity through the manipulation of tumoral ROS levels in tumor occurrence and development.^29,52,168−172^ Therefore, it represents a promising therapeutic target, and it is crucial to understand its involvement in different cancer models.

Despite the field of NOX5 biology still being in its infancy, it has been proven that NOX5 expression and activity are increased in gastric cancer, malignant melanoma, and breast, prostate, and esophageal cancers.^173−175^ Different pathways are implicated in these NOX5-ROS-dependent processes, showing an increased expression of NOX5 connected to signaling molecules like MAP kinases and transcription factors (i.e., p53 and β-catenin).^173,176^ NOX5 has also been shown to be related to the sensitivity of cancer cells to chemotherapeutic drugs such as cisplatin. Specifically, skin, breast, and lung cancer cells treated with this chemotherapy drug rise in ROS-mediated cancer cell death.^174^

While several reports demonstrate the role of NOX1–5 in the tumorigenesis of various tissues, little is known about the DUOXs. DUOX1 expression was found to be lower in liver cancer cell lines in comparison to nontumor tissues.^177,178^ Moreover, DUOX1 expression was correlated to genes able to inhibit tumor progression, and patients with DUOX1 overexpression presented overall survival when compared with those with low expression of the enzyme, thus suggesting that DUOX1 expression could be a prognostic tool for patients with liver tumors.^177,178^ A decreased expression of DUOX1 and DUOX2 was also identified in lung cancer, correlated to hypermethylation of CpG-rich promoter regions of DUOX genes. On the other hand, the reintroduction of functional DUOX1 into lung cancer cell lines increased cell migration, without interfering with cell proliferation.^179^ Thus, the loss of DUOX1 seems to be strongly connected to an invasive metastatic phenotype.

## NOX Inhibitors

The wide-ranging role of ROS in several physiopathological processes has prompted the scientific community to target the different components involved in their production to overcome the redox imbalance often underlying disease states.^180^ Nevertheless, low or basal levels of ROS are required under proper physiological conditions. In this scenario, the selective inhibition of NOX enzymes, which have the sole function of producing ROS, has become an attractive therapeutic target to treat specific diseases.^181^ Recently, the World Health Organization coined the term “naxib” (NADPH oxidase inhibitors) to define a new therapeutic class, thus corroborating the helpfulness of NOX inhibitors.^182^ However, despite the progress made, the identification of suitable NOX inhibitors is still far from being fully achieved. As a matter of fact, most of the reported compounds are pan-NOX inhibitors, lacking selectivity within the NOX members. Moreover, some compounds are not direct NOX inhibitors but affect ROS production interacting with other ROS generating systems, which share some structural features with the NADPH oxidases.

**Figure 7:**
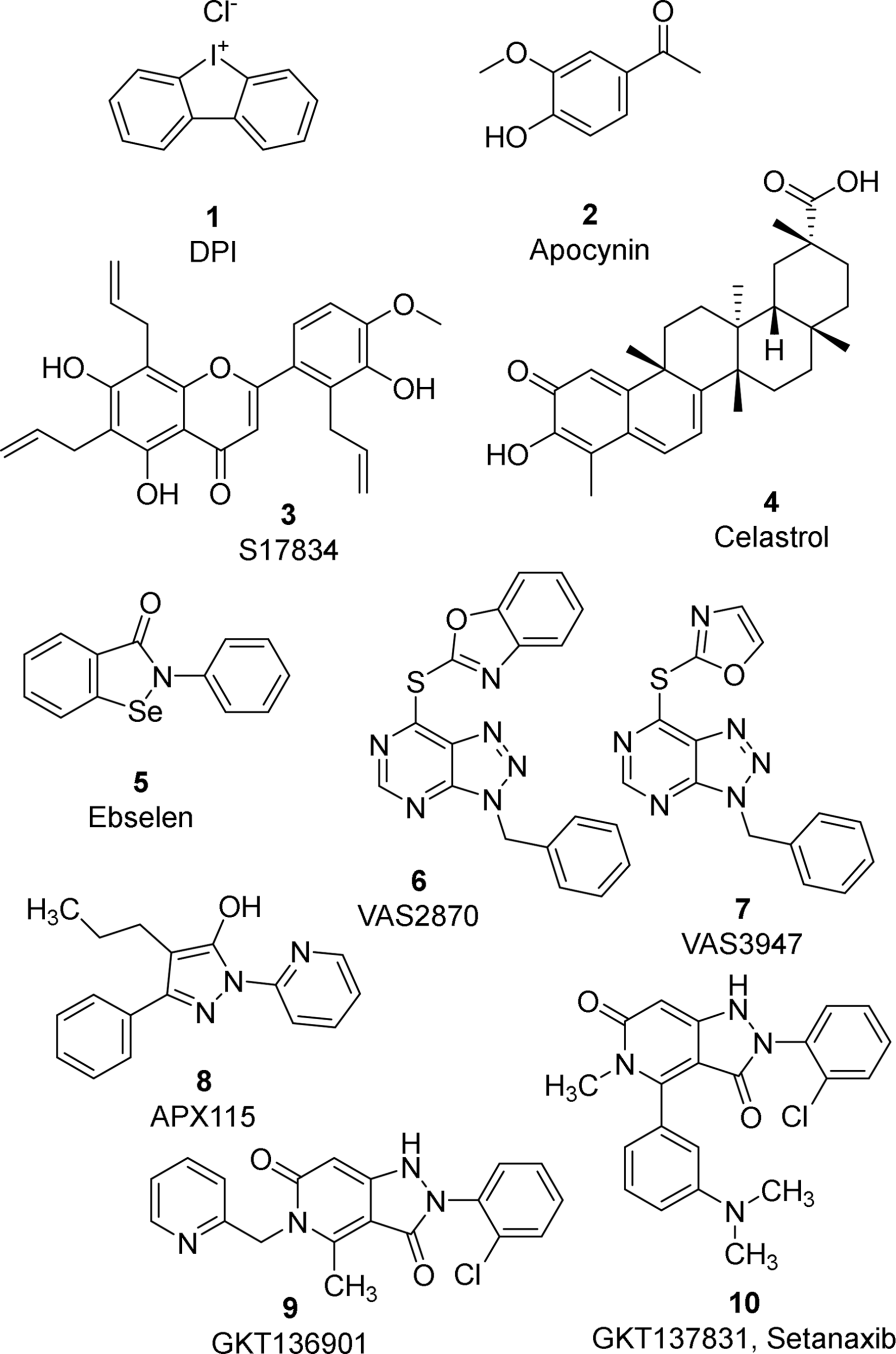
Compound **1** (DPI), **2** (apocynin), and selected pan-NOX inhibitors (**3**–**10**).

The first small molecules used as NOX inhibitors were diphenyleneidonium (DPI, **1**) and apocynin (**2**), as depicted in Figure 7. Compound **1** inhibits all NOX isoforms (IC_50_ of 0.24, 0.10, 0.09, and 0.02 μM for NOX1, NOX2, NOX4, and NOX5, respectively) acting as an uncompetitive inhibitor of flavoproteins.^183^ In addition, **1** reacts with the reduced trans-membrane domain, probably interacting with the heme group through a σ-coordination complex between a phenyl ring of the compound and the iron.^184^

Unfortunately, the binding with flavoproteins allows this compound to inhibit other enzymes, including NOS,^185^ xanthine oxidase,^184^ and cytochrome P450,^186^ as well as interfere with the mitochondrial respiratory chain,^187^ thus hampering its use as a NOX pharmacological tool.

Compound **2** (also known as acetovanillone), extracted from *Picrorhiza kurroa* (kutki, a Himalayan perennial herb used in ethnomedicine), was initially reported as a NOX inhibitor due to its ability to interfere with the intracellular translocation of the cytosolic p47_phox_ and p67_phox_ subunits to the membrane at a concentration of 300 μM. Upon oxidation by myeloperoxidase and activated neutrophils, the compound is converted to the dimeric and trimeric active forms. Moreover, the radical species formed could also be capable of interacting with the thiol groups of the NOX subunits, leading to NOX inactivation.^188,189^ Despite its rather long-term use as an NOX inhibitor, its reliability is rather doubtful, mainly due to direct antioxidant and off-target effects. In fact, it has been shown that the compound failed to inhibit ROS generation in cells overexpressing NOX1, NOX2, and NOX4, acting as an antioxidant scavenger rather than a NOX inhibitor in endothelial and vascular smooth muscle cells.^190^ Moreover, the compound seems to inhibit phagocyte NADPH oxidase but also stimulate ROS production in nonphagocyte cells.^191^ Several off-targets of compound **2** and the related dimeric compound have been reported, including Rho kinase and the PI3K/Akt signaling pathway.^192−194^

Over the years, other small molecules have been reported as NOX ligands, mostly acting as pan-NOX inhibitors (**3**–**10**, Figure 7).

In 2001, Cohen and co-workers reported that, at least in part, the effect of compound **3** (S17834), a benzo(*b*)pyran-4-one able to inhibit the stimulation of tumor necrosis factor-α, of mRNA and protein expression in endothelial cells as well as leukocyte adherence mechanisms, is due to NOX inhibition. Specifically, compound **3** reduced NOX activity in endothelial cell membrane fractions in a concentration-dependent manner and without affecting superoxide production by xanthine oxidase in a cell-free system.^195^ This effect suggested a direct interaction of **3** with the NOXs component, although data on selectivity toward individual isoforms have not been reported. Moreover, the ability of **3** to activate the adenosine monophosphate-activated protein kinase (AMPK) more potently than NOXs has also been shown.^196^

In 2011, Jaquet and co-workers reported **4** (Celastrol) as a NOXs inhibitor.^183^ This bioactive compound, extracted from *Tripterygium wilfordii* (léi go̅ng téng or thunder duke vine, a medicinal plant used in traditional Chinese medicine in immunological diseases), showed a good degree of activity and selectivity for NOX1 and NOX2 (IC_50_ of 0.41 and 0.59 μM, respectively) over NOX4 and NOX5 (IC_50_ of 2.79 and 3.13 μM, respectively). The authors justified this selectivity by considering the mechanism of action; although further studies are needed, compound **4** presumably reacts covalently with the cysteine residues of p47_phox_ and modifies it allosterically, preventing association with the p22_phox_ subunit.^183^ This hypothesis is supported by the lower activity on NOX4 and NOX5, whose activation is independent of this subunit, although it suggests a more complex mode of action. Nevertheless, **4** showed broad activity also on other targets,^197−199^ complicating the interpretation of the specific role in NOXs inhibition. Compound **5** (Ebselen) is a seleno-indoline-like compound able to reduce H_2_O_2_ and other hydroperoxides through its glutathione peroxidase catalytic activity.^200^ In 2012, Lambeth and co-workers reported the compound also as an inhibitor of NOX2 (IC_50_ of 0.3 μM, EC_50_ of 0.5 μM) with cellular activity also on NOX1 and moderately on NOX5 (EC_50_ of 0.15 μM and 0.70 μM, respectively).^201^ Although it was initially suggested that the compound could inhibit the interaction between p47_phox_ and p22_phox_, thus preventing the activation of NOX2,^201^ Bach and co-workers disclosed a different mode of action. In fact, the authors demonstrated that the compound establishes a covalent interaction with a cysteine residue of p47_phox_, leading to destabilization and aggregation of this subunit, which is thus unable to interact with p22_phox_ forming the active enzymatic complex.^202^ This mechanism is likely associated with nonspecific effects, as suggested by the activity of **5** toward different targets, thus hindering its use as a NOX chemical probe.^203,204^

A screening approach for the development of NOX2 inhibitors yielded the triazolo pyrimidine-based compound **6** (VAS2870) capable of inhibiting all cellular NOX isoforms except NOX3 (IC_50_ of 0.5, 0.1, >50, 6.2, 2.1, 0.7, and 2.7 μM for NOX1, NOX2, NOX3, NOX4, NOX5, DUOX1, and DUOX2, respectively), despite being previously discovered as a NOX2 selective inhibitor.^187,205^ The related compound **7** (VAS3947) overcomes the low solubility of the parent compound but shows a similar NOX inhibition profile.^206^ Compound **6** has shown promising beneficial effects in preclinical disease models such as thrombosis, neurodegeneration, and cancer. However, in addition to the lack of selectivity for individual NOX protein isoforms, the utility of compound **6** is compromised by some off-target effects, mainly due to its ability to alkylate thiols by nucleophilic substitution involving the triazolo pyrimidine core. For example, it has been demonstrated that this derivative induced a thioalkylation of the cysteine residues of the ryanodine Ca^2+^ receptor channel, also interfering with its physiological regulation by nitric oxide.^207^ On the basis of these findings, in 2020, Mattevi and co-workers investigated the reactivity of VAS compounds toward cysteine residues of the dehydrogenase domain of NOXs. Specifically, ESI-MS analysis showed the ability of the benzyl-triazolopyrimidine moiety to alkylate a conserved active-site cysteine, thus demonstrating their covalent binding to NOX5.^184^ Recently, Lin and co-workers found that VAS compounds can also act on the downstream PKC signaling pathway through an NOX-independent mechanism. This activity resulted in the inhibition of platelet aggregation, granule release, calcium mobilization, and GPIIb/IIIa activation. In addition, these compounds prevented thrombus formation in mice, without interfering with normal hemostasis.^208^

In 2016, the pyrazole compound **8** (APX-115 also known as Ewha-18278) was reported as a pan-NOX inhibitor (*K*_*i*_ of 1.08, 0.57, and 0.63 μM for NOX1, NOX2, and NOX4, respectively) with no activity on xanthine oxidase or glucose oxidase. The compound showed good potential in the treatment of osteoporosis, since oral administration in mice recovered bone mineral density, trabecular bone volume, and length, number, and thickness.^209^ In addition, **8** improved insulin resistance in diabetic mice and showed a renal protective effect in both type 1 and type 2 diabetes.^210,211^ In 2020, Chung and co-workers evaluated the effect of compound **8** in NOX5 transgenic mice, demonstrating its activity on this isoform as well as its potential for the treatment of diabetic nephropathy.^212^

In 2010, a high-throughput screening campaign followed by structure–activity relationship (SAR) investigation led to the development of **9** (GKT136901), a pyrazolopyridinedione compound claimed to be a preferential inhibitor of NOX1 and NOX4 isoforms (*K*_*i*_ of 160 and 165 nM, respectively) compared to NOX2 (*K*_*i*_ of 1530 nM).^213^ Subsequent optimization led to compound **10** (GKT137831, also known as Setanaxib), which showed a similar selectivity profile (*K*_*i*_ of 140 and 110 nM for NOX1 and NOX4, respectively) with 15-fold less potency on NOX2 (*K*_*i*_ of 1750 nM) and 3-fold less potency on NOX5 (*K*_*i*_ of 410 nM).^214^ Both compounds showed good pharmacokinetic properties and proved to be useful in several animal models. For example, compound **9** showed renoprotective effects in a mouse model of type 2 diabetes, while compound **10** proved to be helpful in preventing hypertensive cardiac remodeling in hypertensive rats induced by abdominal artery coarctation.^215,216^ In October 2013, compound **10** entered a clinical trial to evaluate its efficacy in oral administration in type 2 diabetes patients with maximal inhibition of the renin-angiotensin-aldosterone system and residual albuminuria.^217^ The study concluded in March 2015, but the results are still not available. Currently, compound **10** is being evaluated in two different clinical trials in patients with primary biliary cholangitis (PBC) and liver stiffness,^218^ as well as in patients with idiopathic pulmonary fibrosis.^219^ Despite these interesting results, compound **10** is a selective scavenger of peroxynitrite and hydrogen peroxide as well as ROS.^187,220^ In addition, a recent study has shown the compound to be an interferent in several assays evaluating its activity on NOX proteins, raising questions about the correct interpretation of the data obtained and its actual mode of action and potency.^221^

**Figure 8:**
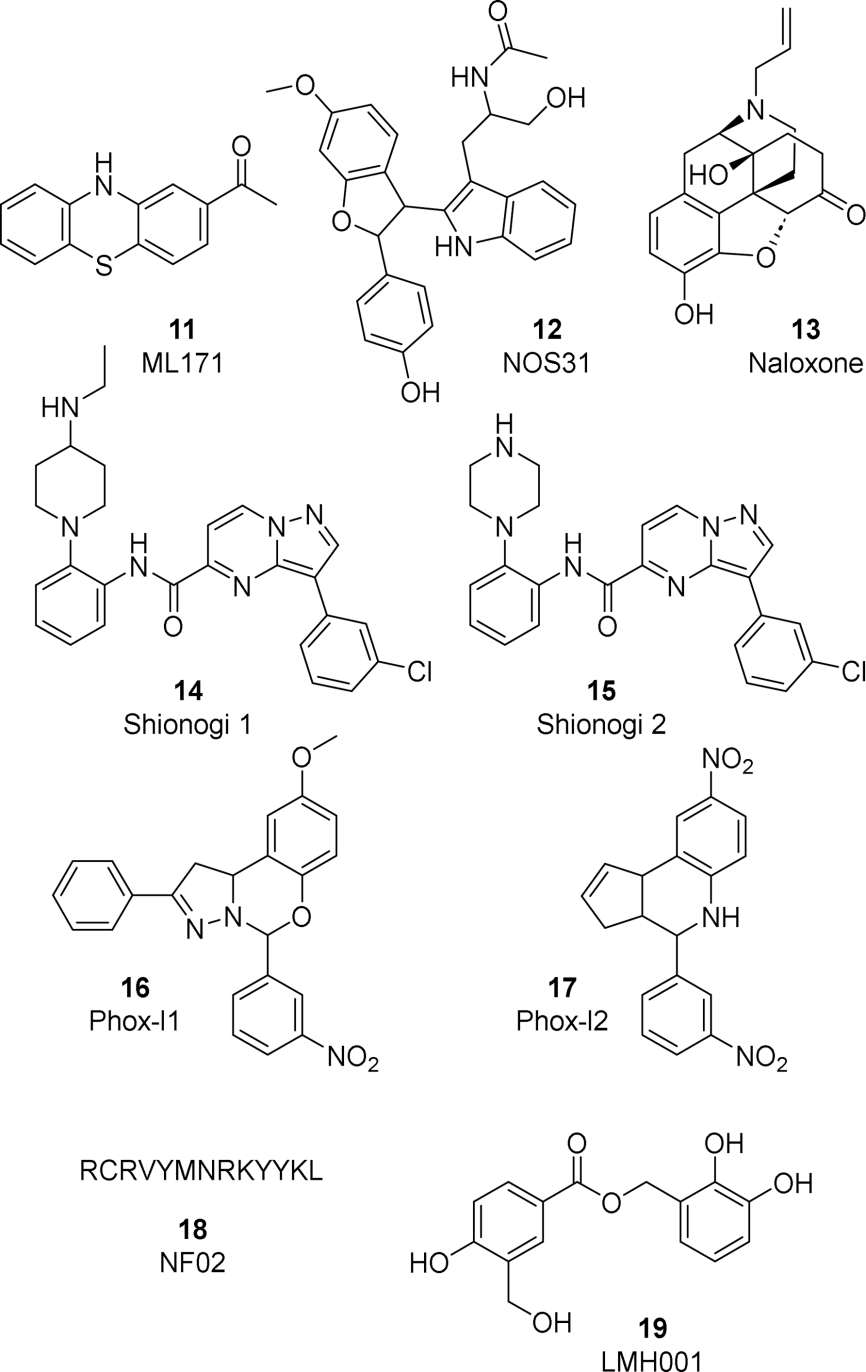
NOX inhibitors with limited or unverified isoform selectivity.

Other compounds have been reported as NOX inhibitors with limited or not completely investigated isoform selectivity (**11**–**19**, Figure 8).

In 2010, the 2-acetylphenothiazine **11** (ML171) was reported as a NOX1 selective inhibitor (IC_50_ of 0.250 μM) with mild activity on other NOXs (IC_50_ of 3–5 μM for NOX2–4) as well as on xanthine oxidase (IC_50_ of 5.50 μM).^222^ Even though it was used in further studies, the reliability of compound **11** as a NOX inhibitor should be carefully evaluated, considering that other studies have demonstrated that the phenothiazine scaffold, a known peroxidase substrate, could interfere with the assay, thus compromising the activity data.^221,223^

A study published in 2018 reported natural compound **12** (NOS31) as a NOX inhibitor secreted from *Streptomyces* sp. The compound showed an interesting activity profile and selectivity on NOX1 (IC_50_ = 2 μM), with at least 14-fold weaker activity on other NOX enzymes. **12** has shown antiproliferative effects in cell lines that upregulate NOX1, such as colon and stomach cancer cells. Although further studies are needed to better characterize its mode of action and selectivity profile, it could be considered a useful tool compound.^224^

In 2012, Hong and co-workers reported compound **13** (Naloxone), an FDA-approved drug for the treatment of opioid overdose, as a NOX2 inhibitor.^225^ In a study aimed at developing new anti-inflammatory drugs for Parkinson’s disease, the authors revealed the compound’s efficacy in preventing dopaminergic neurodegeneration in animal models by inhibiting inflammatory responses. Subsequently, they showed that the compound can inhibit ROS production, thus explicating its anti-inflammatory and neuroprotective effects. Specifically, **13** binds to the catalytic subunit of microglial NOX2 (IC_50_ of 1.96 and 2.52 μM for the (−) and (+) isoforms, respectively) and reduces the translocation of cytosolic subunits to the plasma membrane, thus preventing activation of the enzyme. Naloxone has ROS scavenging properties and is inactive on xanthine oxidase.^225^ Exploiting these mechanisms, a recent work proposed the repositioning of **13** as a neuroprotective therapeutic strategy to reduce stroke severity in opioid abuse. The administration of the opioid antagonist as a pure nanodrug effectively reduces morphine- or oxycodone-induced superoxide production and, overall, could be used as a promising therapeutic strategy for protection against oxidative stress.^226^ Unfortunately, the effects of this inhibitor on other NOX isoforms have not yet been studied. Other interesting inhibitors are the pyrazolo pyrimidine derivatives **14** and **15** (Shionogi 1 and 2, respectively) reported as potent NOX2 inhibitors (IC_50_ of 56 and 99 nM for **14** and **15**, respectively). Their proposed mode of action is based on the inhibition of protein kinase Cβ II (PKCβII), which becomes incapable of performing its task in p47_phox_ translocation. No data are available on the selectivity and the activity of these two inhibitors on other NOX isoforms. Moreover, the compounds should be considered only indirect inhibitors of NOX2, mainly acting on PKCβII.^227^

In an *in silico* screen to identify inhibitors of the Rac1–p67_phox_ interaction, a fundamental step in NOX2 activation, compound **16** (Phox-I1) was identified as a new NOX2 inhibitor. This molecule was able to strongly interact with p67_phox_ and compete with Rac1, leading to NOX2 inactivation. The compound was further optimized in the more soluble derivative **17** (Phox-I2), which inhibits NOX2 (IC_50_ of around 1 μM) with no effect on xanthine oxidase or on cells overexpressing NOX4, according to its independence from Rac1. However, further investigations are needed to assess selectivity on NOX3, NOX5, and DUOX isoforms and mostly on NOX1, whose activity also depends on Rac1.^228^

In 2017, peptide **18** (NF02) was identified in a screening for the identification of NOX1-selective inhibitors. This compound is a 13-amino acid peptide (sequence RCRVYMNRKYYKL) able to significantly lower ROS production in cells at 10 μM, with an IC_50_ value of 16.7 μM. **18** proved to be effective in reducing migration and invasion of colorectal cancer cells. Despite the authors verifying the selectivity of this compound for NOX1 over NOX2, the effects on other NOX isoforms were not reported.^229^

In 2022, based on the crystal structure of p47_phox_ tandem SH3 domains, the catechol ester derivative **19** (LMH001) was designed to bind the exposed SH3 binding pocket and thus inhibit NOX2 activation. In a fluorescence polarization assay, compound **19** was shown to inhibit the p47_phox_/p22_phox_ interaction with an IC_50_ of 0.149 μM and a *K*_*i*_ of 0.054 μM and was able to inhibit AngII-induced endothelial NOX2 activation and superoxide production. In addition, **19** reduced hypertension and aortic wall inflammation in a mouse model of AngII-induced vascular oxidative stress.^84^ However, a following study by Bach and co-workers raised concerns about this compound as a NOX2 inhibitor.^230^ Indeed, **19** was shown to be hydrolyzed in standard aqueous buffer and was not able to inhibit the p47_phox_/p22_phox_ interaction. Moreover, cellular studies for **19** demonstrated a weak NOX2 inhibitory activity (IC_50_ of 54 μM), comparable to that of the catechol hydrolysis product. It is to be noted that catechol containing compounds are flagged as redox cyclers and could lead to assay artifacts.^231^

**Figure 9:**
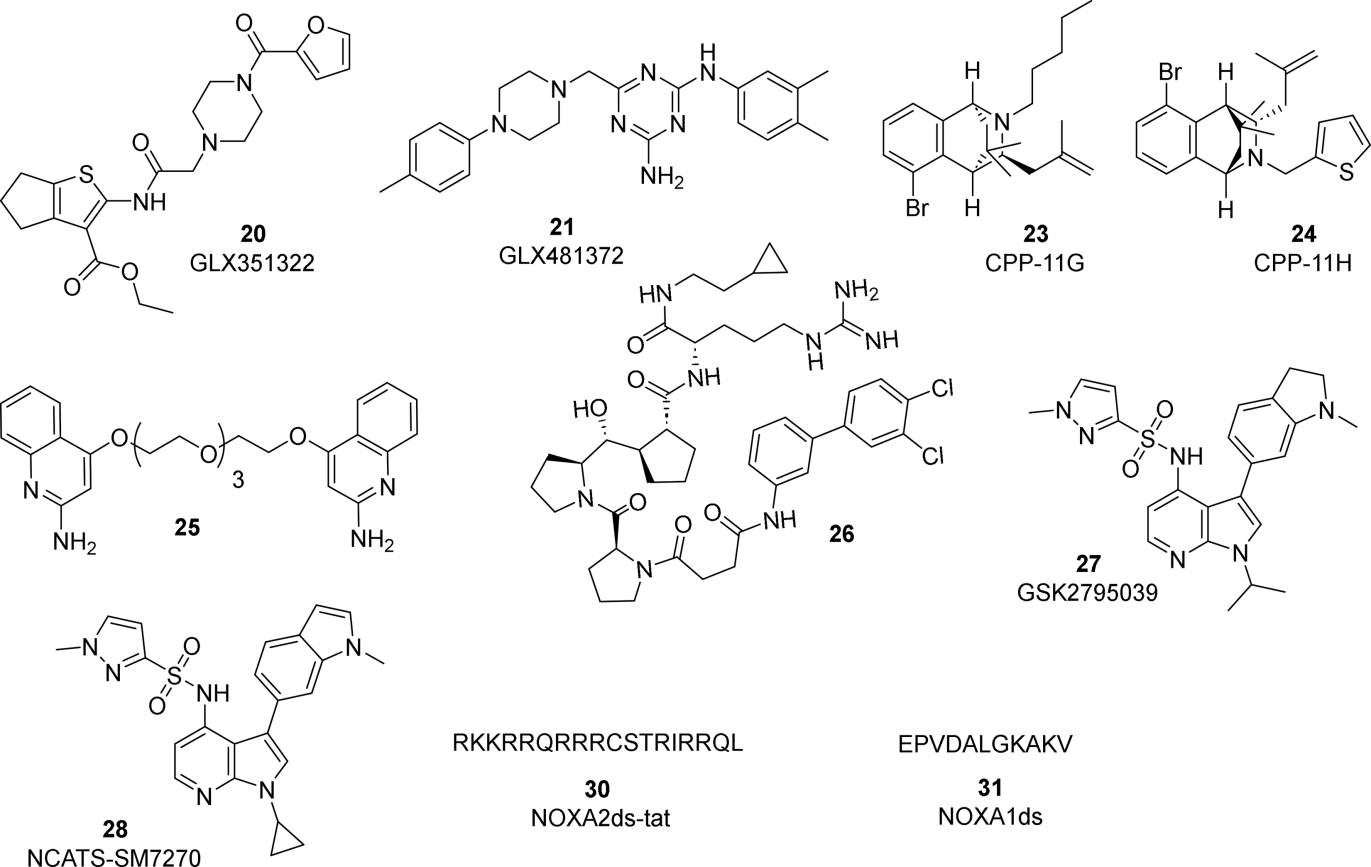
GLX compounds and isoform-selective NOX inhibitors. Structures of compounds **22** and **29** have not yet been disclosed.

Although few inhibitors with enhanced isoform-specificity have been developed (**20**–**31**, Figure 9). The starting point for the development of NOX4-selective ligands was the screening of a library of 40,000 compounds in T-Rex-293 cells with inducible overexpression of NOX4 in order to investigate the role of this enzyme in type 2 diabetes. This led to the identification of compound **20** (GLX351322).^232^ Alongside good solubility, chemical and metabolic stability, and membrane permeability, compound **20** has good selectivity for NOX4 with an order of magnitude lower inhibitory potency against NOX2 (IC_50_ of 5 μM on NOX4 in T-REx-293 cells compared to IC_50_ of 40 μM on NOX2 in *h*PBMC cells). This molecule was able to partially prevent ROS production, islet death, and insulin release caused by high glucose levels. The authors showed that NOX4 inhibition *in vivo* affected pancreatic islets and not the peripheral tissues targeted by insulin, speculating that this could also be explained by a low degree of NOX2 inhibition.^232^ Unfortunately, further studies revealed that **20** inhibited NOX1 and NOX5 with similar efficacy as NOX4.^233^ The same research group undertook a SAR campaign on identified hits with selectivity for NOX4 over NOX2, developing two other NOX inhibitors: compound **21** (GLX481372) and compound **22** (GLX7013114, structure not disclosed).^233^ The first one (**21**) is active in the submicromolar range against both NOX4 and NOX5 isoforms (IC_50_ of 0.68 and 0.57 μM, respectively) and about 10-fold less active against NOX1, NOX2, and NOX3 (IC_50_ of 7, 16, and 3.2 μM, respectively). On the other hand, compound **22** showed good and promising selectivity for NOX4 (IC_50_ of 0.3 μM) with no reported activity against NOX1, NOX2, NOX3, and NOX5, as well as against xanthine oxidase or glucose oxidase. The authors hypothesized that the selectivity gained on NOX4 arises from targeting a unique region of the enzyme, nonconserved between other NOXs isoforms. This compound showed a good potential in protecting human islet cells from hyperglycemia-induced death. In addition, the derivative prevented the upregulation of mesenchymal genes induced by transforming growth factor β (TGFβ), highlighting the role of NOX4 in the regulation of these genes involved in the epithelial–mesenchymal transition of the lens.^234^ The good selectivity and the reported absence of off-target effects make this compound a valuable tool for deepening the biological role of NOX4.

On the other hand, a widely investigated strategy to obtain putative NOX2 selective ligands relies on the inhibition of protein–protein interaction between the p47_phox_ and p22_phox_, which prevents the assembly and the activation of the NOX2 complex. In this context, two bridged tetrahydroisoquinolines **23** and **24** (CPP-11G and CPP-11H, respectively) were identified as selective NOX2 inhibitors (IC_50_ of 20 and 32 μM, respectively) with lower IC_50_ values claimed in a cell-free system (approximately 30 nM), even if these data were not shown in the paper. The compounds displayed no detectable activity against NOX1, NOX4, NOX5, and xanthine oxidase, as well as no ROS scavenging properties. From a structural point of view, the insertion of small substituents such as *n*-pentane and thiophene on the nitrogen atom of the tetrahydroisoquinoline scaffold influenced the selectivity toward NOX2 inhibition.^235^ These compounds hindered p47_phox_ translocation from the cytosol to the plasma membrane, preventing its interaction with the p22 subunit and thus NOX2 activation. By inhibiting NOX2, both compounds were effective in reducing TNFα-stimulated endothelial inflammation and dysfunction in human cells *in vitro* and in mice *in vivo*.^236^ Overall, **23** and **24** have a promising selectivity profile, although a more complete assessment of the IC_50_ values is necessary.

Recently, a screening of 2,500 fragments using different biophysical techniques allowed the identification of two promising quinoline-containing hits able to bind the SH3 domain within the p47_phox_ subunit with *K*_D_ of approximately 400–600 μM. Structural studies revealed that each one of these two fragments binds to two separate sites at the extended conformation of the p47_phox_ subunit. Thus, the authors concluded that the observed inhibition could be due to the ability of the compounds to reduce the level of binding of p22_phox_ or to the resulting stabilization of the extended conformation of p47_phox_, which is consequently unable to assume the correct conformation to create the binding pocket to p22_phox_. To further improve the affinity and support this binding model, three homodimeric compounds based on the two fragments and containing a PEG-based linker were synthesized and tested. Among them, compound **25** was found to be able to inhibit the p22_phox_–p47_phox_ protein–protein interaction by binding to both SH3 domains of p47_phox_ (*K*_*i*_ = 20 μM). Despite being interesting, further optimizations are needed to obtain affinities relevant for more advanced biological studies.^202^

In 2022, a structure-based design strategy allowed the development of the peptide-derived triproline mimetic compound **26**, which mimics an identified hot spot sequence (PPP) involved in the most important interactions with the p47_phox_ subunit. This derivative showed a submicromolar *in vitro* affinity (*K*_D_ of 0.312 μM) against the binding of the two subunits and could be further optimized to improve the affinity and drug-likeness.^237^

In 2015, Glaxo Smith Kline, in collaboration with the University of Geneve, carried out a high-throughput screening, followed by a lead optimization campaign, from which the 7-azaindole **27** (GSK2795039) emerged.^238^ The compound is reported as a competitive and selective inhibitor of NOX2 (pIC_50_ value is 5.5 to 6.5, depending on assay performed) with more than 100-fold selectivity over xanthine oxidase and only 50% inhibition of eNOS at 100 μM. The selectivity for NOX2 over other NOX isoforms was evaluated in two different cellular assays, which, interestingly, gave two opposites outcomes. Nevertheless, the authors confirmed NOX2 selectivity, attributing the inconsistent results to an interference in one of the assays used, related to the weak reducing activity of the compound. It was also demonstrated that **27** does not react with thiol residues in the catalytic domain of NOX2 but competes for its NADPH binding site, showing lower inhibitory potency as NADPH concentrations increase.^238,239^ The compound was found to be effective in reducing ROS production in microglia cells, supporting the hypothesis that iron utilizes NOX-produced superoxide and hydrogen peroxide to increase ROS production, consequently contributing to oxidative stress in neurodegenerative diseases.^240^ Moreover, the inhibitor performed well in the mouse model of traumatic brain injury (TBI), reducing NOX2 expression and attenuating TBI-induced neurological deficits.^241^ Despite its efficacy as an NOX2 inhibitor, **27** has poor bioavailability and moderate-to-high clearance. A recent work analyzed the compound’s metabolic pathways in microsomal and cytosolic fractions of mouse, rat, and human liver.^242^ The information obtained guided further structural optimization to improve the pharmacokinetic characteristics. Specifically, a group of different analogues of **27** was designed and synthesized, and their inhibitory activity against NOX2 was evaluated.^243^ Compound **28** (NCATS-SM7270) emerged as the best candidate, showing a 2-fold higher potency against NOX2 than parent compound **27** (IC_50_ of 2.1 and 3.94 μM, respectively, in PMNs extracted from mouse granulocytes). In addition, the compound displayed improved permeability, solubility, and half-life in rat microsomes. From a structural point of view, the compound retains the 7-azindole core of compound **27** but has a different substitution pattern at both position 1 (the isopropyl substituent of **27** has been replaced by a cyclopropyl group) and position 3 (the methylindoline of **27** has been replaced by a methylindole). The authors also analyzed the selectivity of the compound against other NOXs, showing that there is no detectable activity against NOX3 and NOX4 and only marginal activity against NOX1 and NOX5 at the highest dose tested. Interestingly, the authors were unable to detect differences in the activity of **27** in the group of NOXs tested, even though the compound maintains the same potency as reported on NOX2. In primary mouse neutrophils, the compound was found less potent than **27** (IC_50_ of 4.8 and 2.17 μM, respectively), suggesting that it might be less active in mouse. Finally, transcranial administration of **28** resulted in a good reduction of cortical cell death in a murine mild TBI model, with no beneficial effects in NOX2 knockout mice.^243^

In 2019, Genkyotex developed compound **29** (GKT771, structure not disclosed) as a selective NOX1 inhibitor (*K*_*i*_ of 60 nM) with a high degree of selectivity toward NOX4 (*K*_*i*_ of 4 nM) and no activity against all other NOX isoforms, as well as xanthine oxidase and glucose oxidase and no scavenging properties. The activity of the compound was investigated both in a colon carcinoma and a melanoma mouse model. Overall, it elicited immunomodulatory effects essential for its antitumor activity.^244^

Also, two peptide-derived compounds have been described as selective NOX inhibitors. In 2001, Pagano’s group developed compound **30** (NOXA2ds-tat), an 18-amino acid peptide (sequence RKKRRQRRRCSTRIRRQL) developed from a 9-amino acid peptide of the NOX2 intracellular B-loop sequence (NOX2ds) that allows the binding to p47_phox_ and from a chimeric peptide containing 9 amino acids of the HIV-TAT sequence (named *tat*) that allows cell permeation.^245^ The NOXA2ds sequence permitted a strong interaction of compound **30** (IC_50_ of 0.74 μM on NOX2) with the p47_phox_ subunit, thus preventing the assembly and the activation of the NOX2 complex. Considering the high homology in the catalytic subunit B-loop sequences of NOX2, NOX1, and NOX4 and their similar activation, the authors studied the specificity of NOX2ds-tat on these isoforms as well. The data obtained clearly showed that **30** is unable to inhibit NOX1 and NOX4 and can be considered a selective inhibitor of NOX2.^246^ Although the compound, as a peptide, has low oral bioavailability, its parenteral administration in mice and human resistance artery smooth muscle cells reduced blood pressure and AngII-induced superoxide production, with no notable adverse reactions at the tested concentrations.^245^ In addition, the peptide has been successfully used to investigate the role of NOX2 in ischemia/reperfusion injury, vascular compensation to oxidative stress-associated arterial occlusion, and atherosclerosis, resulting in regression of atheromatous plaques in apolipoprotein E-deficient mice.^247,248^

In 2013, the same research group designed peptide **31** (NOXA1ds), whose structure is based on a short sequence of the NOX1 activating subunit NOXA1 (sequence EPVDALGKAKV).^249^ Through this sequence, the compound is able to disrupt NOX1-NOXA1 association, strongly inhibiting NOX1 activity (IC_50_ of 19 and 100 nM for cell lysates and whole HT29 cells, respectively). Considering the homology between NOXA1 and the NOX2 activating subunit p67_phox_, the authors selected a portion of the sequence in which 46% of the amino acids are dissimilar between p67_phox_ and NOXA1 in order to obtain NOX1 specificity. In fact, **31** was found to be selective for NOX1 over NOX2, NOX4, and NOX5 as well as inactive against xanthine oxidase and was used to investigate the role of NOX1 in a different type of hypertension, pulmonary arterial hypertension.^250−252^

## Biological Assays for the Identification of NOX Inhibitors

The evaluation of the activity of potential NOX inhibitors is generally performed by applying different enzymatic assays based on the measurement of substrates (i.e., NADPH or O_2_) and/or reactive oxygen species (i.e., superoxide anion and H_2_O_2_) that are consumed and/or generated by NOX proteins.

Absorption, fluorescence spectroscopy, and chemiluminescence are the usual readouts of the main NOX assay formats, which are commonly distinguished as cell-based and cell-free. Cell-based assays rely on the use of purified neutrophils, neutrophil-like cell lines HL60, and PLB cells transfected or transduced with the specific NOX isoform. Although they represent the optimal systems to study and characterize the mechanism of action of potential NOX inhibitors, some drawbacks must be considered. The simultaneous presence of multiple NOX isoforms at different cellular locations and the production of ROS from other sources as well as the existence of endogenous enzymatic and nonenzymatic antioxidants rapidly reacting with superoxide anion and H_2_O_2_ make the univocal attribution of observed effects to selective NOX inhibition challenging.^238,253^

To overcome all of these issues, cell-free NOX systems (also known as “broken cells” or *in vitro* systems) have been developed. They usually consist of a phagocyte membrane (native or solubilized), a mixture of oxidase components as purified or relipidated recombinant proteins (flavocytochrome b_558_, p47_phox_, p67_phox_, RAC1/2), the substrate NADPH, the target oxygen, and an *in vitro* activator, commonly referred to as an anionic amphiphile (e.g., long chain unsaturated fatty acids or sodium/lithium dodecyl sulfates). Recently, some simplified systems have been described. Specifically, the three individual cytosolic oxidase components are replaced by a p47_phox_-p67_phox_-RAC chimera or by a prenylated RAC component, both capable of oxidase activation also in absence of the activator.^254,255^ Compared to the cell-based system, the cell-free system allows the precise quantification and reproducibility of the reaction products (superoxide anion and H_2_O_2_), the easy generation of dose–response curves, and the calculation of kinetic parameters as well as represents an excellent potential for high-throughput screening and automation. Moreover, due to the ability to distinguish the oxidase assembly phase (NOX cytosolic component interaction) from the catalytic phase (electron flow from NADPH to O_2_), this assay format is useful for defining the step interfered by the inhibitor.

Some of the most used methods suitable for cell-free and cell-based assays are briefly described hereafter.

### Cytochrome c Reduction Assay

This colorimetric assay is based on the measurement in biological samples, in the presence of NADPH, of NOX-generated superoxide, which is responsible for the reduction of cytochrome c. The absorption spectrum of cytochrome c is dependent on its oxidation/reduction state. In the reduction state, an absorption peak at 550 nm can be observed, and increasing absorbance can be monitored spectrophotometrically.^256^

### MCLA Assay

Methyl Cypridina luciferin analogue (MCLA) is a specific chemiluminescent probe that is commonly used to evaluate superoxide formation, as this reagent is highly sensitive to superoxide. In fact, MCLA reacts with superoxide generating a chemiluminescent signal, which can be detected by using a suitable plate reader.^257^

### Amplex Red/Peroxidase Assay

The amplex red reagent is a fluorogenic substrate for peroxidase and is used to probe hydrogen peroxide. This reagent, in the presence of horseradish peroxidase (HRP), reacts with H_2_O_2_ with a 1:1 stoichiometry to generate resorufin, a highly fluorescent product, which can be determined by the use of a fluorimeter.^258^

### CBA Assay

Coumarin boronic acid (CBA) is a fluorogenic reagent that is used for the determination of hydrogen peroxide. CBA reacts with H_2_O_2_ with a 1:1 stoichiometry to generate the fluorescent 7-hydroxy-coumarin, which can be quantified by the use of a fluorimeter.^259^

The advantages of all these assays rely on the speediness and ease of experimental preparation and data analysis. Nevertheless, all of them are highly prone to interference since inhibitors can show ROS-scavenging properties or react with any reagent of the assays, thus altering the reliability of data obtained and furnishing false positives.

## Monitoring of Oxygen and NADPH Consumption

In addition to the above-mentioned assays that monitor the formation of ROS species, the activity of NOXs can also be evaluated by the assessment of the rate of oxygen and NADPH consumption. Differently from the oxygen consumption that can be measured in intact cells using a Seahorse XF96 extracellular flux analyzer (Agilent Technologies), NADPH depletion is measured only in cell-free assays considering its involvement in several biological pathways. NADPH is added to the samples as a bolus, and the rates of consumption are easily monitored by a spectrophotometric analysis at the wavelength of 340 nm.^238,260^

The quantitative assessment of substrate consumption is advantageous as no probes are needed, reducing the common artifacts related to their use. Moreover, due to the recent developments in monitoring oxygen consumption rates in real-time and in a multiwell plate format, a discrete throughput is achievable.

## Fluorescence Polarization Assay

Considering all the limitations of the assays based on the measurement of ROS species, in 2012, a fluorescence polarization (FP) assay was developed, which detects the formation of the protein complex necessary for NOX2 activity.^201^ As mentioned above, activation of NOX2 requires the association between p22_phox_ and p47_phox_ subunits.^261^ The FP assay was designed using a synthetic peptide of p22_phox_ containing PRD labeled with rhodamine dye and a GST-tagged bis-SH3 domain of p47_phox_. When the two subunits are in the proximity, they bind each other, and a high fluorescence polarization signal is recorded. When an inhibitor is added to the assay mixture, the binding of the two subunits is prevented, resulting in a decrease of FP signal. This competition assay is well suited for high-throughput screening considering the low materials required; however, the identified hits need to be confirmed in functional assays in order to prove the inhibiting activity on NOX2.

## Conclusions and Future Perspective

NOX proteins represent appealing targets for drug discovery, considering their involvement in the development and maintenance of several pathological conditions.

One of the challenges in developing NOX inhibitors is their specificity. NOX enzymes are involved in various physiological processes, and inhibiting them may cause unintended side effects. Therefore, it is crucial to develop NOX inhibitors that selectively target the specific isoforms of NOX involved in the pathogenesis of a particular disease. Additionally, some isoforms of NOX may have different functions in different tissues, so it is essential to also consider the tissue-specificity of NOX inhibitors.

However, considering the features of this class of enzymes and their mechanism of action, the identification of selective NOX inhibitors is quite challenging. In fact, as mentioned above, the inhibition of NOXs can be achieved exploiting different mechanisms, which range from the inhibition of subunit assembly to the competition with substrates or the scavenge of superoxide/hydrogen peroxide, all strategies susceptible to low specificity of action.

Indeed, over the past years, several putative NOXs inhibitors have been developed through different drug discovery strategies, and some of them have confirmed their potential therapeutic effects in *in vitro* or *in vivo* models of diseases associated with oxidative stress. Yet, many of the reported compounds are pan-NOX inhibitors, lacking in selectivity within NOX members, or are indirect NOX inhibitors, affecting ROS production by interacting with other ROS generating systems which share some structural features with the NADPH oxidases. For most of the others, selectivity data are not available.

Moreover, most (if not all) small-molecule inhibitors of NOX family members developed to date target intracellular regions, where subtype selectivity has been difficult to achieve. In any case, very little information (if any) is available on the binding mode of the inhibitors thus far identified.

Even if no cocrystal structure of NOX enzymes in complex with ligand has been reported to date, docking and molecular dynamics studies using the available structures of TM and DH domains can be used to virtually screen a large part of the chemical space to identify potential ligands and/or provide structural hints on the binding mode, thus informing the development of better inhibitors. The applicability of the approach could be made even broader by including three-dimensional modeled structures predicted by AlphaFold^262^ in combination with virtual mutagenesis studies.

An example of this approach was recently applied to NOX1 and led to the identification of a promising hit.^263^

Noteworthy, this approach could also help to design targeted covalent inhibitors that in turn could increase selectivity and, by using the structures predicted by AlphaFold, could be extended to the other subunits of the NOX complexes and used to design inhibitors based on peptide sequences from protein–protein interfaces and, then, small molecule peptidomimetics.

The recent elucidation of the NOX2 core structure in complex with the anti-NOX2 antibody 7G5 demonstrated that the extracellular cap of NOX2 can be targeted by a selective antibody and suggested a mechanism by which the ECLs may allosterically modulate NOX2 function, thus paving the way for the development of allosteric inhibitors.^28^ To this aim, again, *in silico* studies could provide an invaluable tool to computationally design cyclic peptides derived, for example, from an antibody loop, as already done in other research fields.^264^

Other alternative, underexplored target for the discovery of NOX inhibitors is glycans, at least for a few isoforms. In fact, as mentioned above, for NOX2, cell surface recognition exclusively occurs in the complex *N*-glycan-carrying form. Similarly, *N*-glycosylation plays an important role in the regulation of the activity of the other isoforms (with the exception of NOX5).^265^ Hence, strategies aimed at designing glycomimetics able to block specific lectin–carbohydrate interactions could be pursued in future drug discovery campaigns.

Alongside the identification of potential hits, another important issue is to discriminate between “real inhibitors” and assay interference compounds. In this regard, several cell-based and cell-free enzymatic assays for the evaluation of NOXs activity are available. However, most of these methods are subjected to artifacts because of the potential interference of compounds with assay reagents, leading to false positive readout. As a matter of fact and as mentioned above, many compounds claimed to be potent NOXs inhibitors and also evaluated in *in vivo* models of oxidative stress later proved to be false positives.

Therefore, medicinal chemistry efforts should accomplish a full biochemical characterization of the identified inhibitors in order to provide valuable chemical probes to further elucidate the physiopathological role of the NOX enzymes. To overcome the limits of the available screening methods, an appropriate approach should combine the use of different inhibition assays with the evaluation of the direct binding of potential inhibitors with NOX isoforms, applying different assays.^266,267^ This multiple approach allows one to define genuine inhibition of the proteins caused by functional binding of compounds, avoiding molecules responsible for nonspecific inhibition. Additionally, the application of orthogonal biophysical methods to evaluate interactions is a widely reported strategy,^268^ which could help in the validation of obtained binding data, allowing the identification of real and robust inhibitors. An example of a successful application of this combined approach has been recently reported by Mattevi and co-workers,^184^ who demonstrated that it can be exploited as a model to continue the research of inhibitors in this field. Similarly, Bach and co-workers used a combination of biophysical techniques to validate, characterize, and evolve hits identified through a fragment-based approach.^202^

Importantly, compounds featuring redox-active scaffolds and other frequent hitters should already be excluded from further optimization during the early stages of hit discovery campaigns or at least taken into consideration as potential sources of artifacts, preferring instead to pursue hits with tractable mechanisms of action.

In conclusion, the identification of real and selective NOXs inhibitors requires the application of a wide and complex workflow, which includes the evaluation of both the inhibiting effect through enzymatic assays and the binding of the compounds to NOX proteins. Thanks to the application of this strategy and also considering new emerging targets and techniques, it will be possible to increase the number of available validated selective inhibitors for NOX enzymes, which to date are in a limited number, and consequently to expand the knowledge in this field.

## Abbreviations

CCR2: CC chemokine receptor 2
CCL2: CC chemokine ligand 2
CCR5: CC chemokine receptor 5
TLC: thin layer chromatography
